# CMTM family proteins 1–8: roles in cancer biological processes and potential clinical value

**DOI:** 10.20892/j.issn.2095-3941.2020.0032

**Published:** 2020-08-15

**Authors:** Jie Wu, Lan Li, Siyi Wu, Bin Xu

**Affiliations:** ^1^Cancer Center, Renmin Hospital of Wuhan University, Wuhan 430060, China

**Keywords:** CMTM family, cancer, cell cycle, EGFR, EMT, apoptosis, tumor immunity

## Abstract

The CKLF-like MARVEL transmembrane domain containing (CMTM) family of genes comprises CKLF and CMTM1–8 (previously called chemokine-like factor superfamily 1–8, CKLFSF1–8). The CMTM family proteins contain a structurally conserved MAL and related proteins for vesicle trafficking and membrane linking (MARVEL) domain. Dysregulated expression of multiple CMTM family members is a common feature in many human cancer types. CMTM proteins control critical biological processes in cancer development, including growth factor receptor activation and recycling, cell proliferation, apoptosis, metastasis, and immune evasion. Emerging *in vivo* and *in vitro* evidence indicates that the mechanisms of action of most CMTM proteins are complex and multifactorial. This review highlights new findings regarding the roles of CMTM1–8 in cancer, particularly in tumor growth, metastasis, and immune evasion. Additionally, the potential clinical value of CMTMs as novel drug targets or biomarkers is discussed.

## Introduction

Cancer is a highly heterogeneous group of diseases and one of the leading causes of death worldwide. Each tumor type exhibits distinct biological characteristics, clinical features, outcomes, and responses to therapies. Although the mechanisms involved in cancer development have been extensively studied, much remains to be learned about cancer-associated biological regulation. The CKLF-like MARVEL transmembrane domain containing (CMTM) family of genes comprises CKLF and CMTM1–8, and their encoded proteins are structurally characterized as similar to chemokines and members of the transmembrane-4 superfamily (TM4SF). The CMTM family was first cloned and reported in 2001^1^. To date, CMTM family members have been revealed to function as regulators in various diseases, including autoimmune^2^ and cardiovascular diseases^3^. Moreover, recent in-depth studies have indicated that CMTMs also play crucial roles in cancer-associated biological regulation.

CMTM genes have been reported to be differentially expressed between tumor and normal tissue, thus suggesting that CMTMs may actively regulate tumor development in various cancer types^4–10^. The functions of CMTM family proteins in tumor growth, metastasis, and antitumor immunity are well recognized^11–14^. In addition, CMTM family proteins play crucial roles in mediating the clinical characteristics of tumors, including promoting chemotherapeutic resistance in non-small cell lung cancer (NSCLC)^15^, and have prognostic value in multiple cancers^15–18^. In this review, we focus on the CMTM family’s biological effects in tumors and potential clinical applications.

## Structural characteristics of CMTM family gene transcripts and proteins

**Figure 1:**
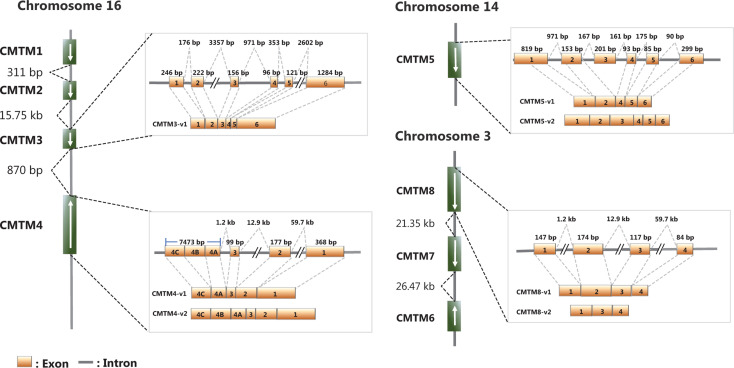
Chromosome locations and genomic structures of the human CMTMs (modified from references 9, 19, 20, 24, and 25). The schematic maps and major splice forms of human CMTM3–CMTM5 and CMTM8 are presented.

CMTM1–8 were first identified in 2003 through analysis combining CKLF2 cDNA and protein sequence analysis with experimental validation^19^. CMTM1 contains a C-c motif and exhibits higher sequence identity with chemokines than do other CMTMs. CMTM8 has the lowest sequence identity with chemokines but has 39.3% amino acid similarity with TM4SF11^19^, and the level of sequence identity between CMTM2–7 and chemokines is intermediate between those of CMTM1 and CMTM8^19^. Thus, proteins encoded by CMTM1–8 have a common feature of structural similarity with classical chemokines and TM4SF. Most CMTM transcripts have multiple alternative splicing forms, but all the resulting protein products contain a MAL and related proteins for vesicle trafficking and membrane linking (MARVEL) domain^19^. Therefore, these proteins were renamed CMTM1–8, or CKLF-like MARVEL transmembrane domain containing 1–8, whereas they were previously called chemokine-like factor superfamily 1–8 (CKLFSF1–8)^20^. The functions of individual CMTM family members in biological processes may depend on the specific alternative splicing isoforms of each transcript^21,22,23^. The genes in the CMTM family form 2 gene clusters. The CMTM1–4 genes form a gene cluster on chromosome 16, whereas CMTM6–8 form the second gene cluster on chromosome 3p22.3 (**Table 1**, **Figure 1**), where many critical tumor suppressor genes are located.

## Expression of CMTM family members

**Figure 2:**
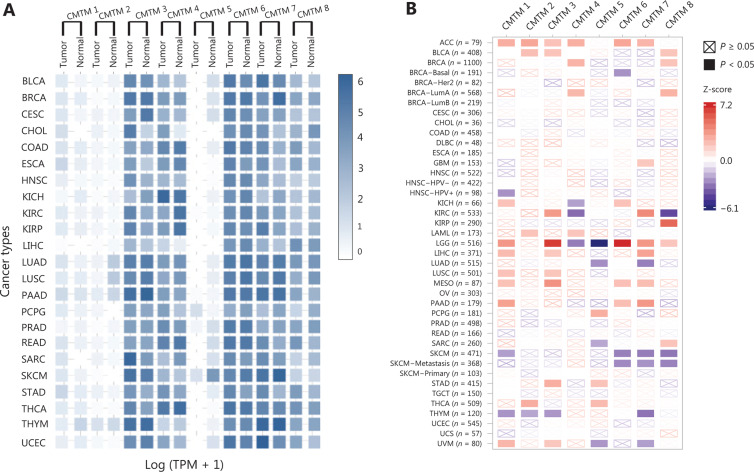
Pan-cancer expression of CMTMs and their prognostic value in TCGA. (A) Expression matrix plots showing gene expression of CMTMs in different cancers. The results were derived from the Gene Expression Profiling Interactive Analysis (GEPIA) database. The density of the color in each block represents the median expression value of a gene in a given tissue, normalized by the maximum median expression value across all blocks. (B) The prognostic value of CMTM gene expression. The results were derived from the Tumor Immune Estimation Resource (TIMER) database. The univariate Cox proportional hazard model was used to evaluate the significance of gene expression outcomes (the gene expression was treated as continuous variable). The heatmap shows the normalized coefficient (Z-score: coefficient/standard error) of the gene in the Cox model. ACC: adrenocortical carcinoma; BLCA: bladder urothelial carcinoma; BRCA: breast invasive carcinoma; CESC: cervical squamous cell carcinoma and endocervical adenocarcinoma; CHOL: cholangial carcinoma; COAD: colon adenocarcinoma; DLBC: lymphoid neoplasm diffuse large B-cell lymphoma; ESCA: esophageal carcinoma; GBM: glioblastoma multiforme; HNSC: head and neck squamous cell carcinoma; KICH: kidney chromophobe; KIRC: kidney renal clear cell carcinoma; KIRP: kidney renal papillary cell carcinoma; LAML: acute myeloid leukemia; LGG: brain lower grade glioma; LIHC: liver hepatocellular carcinoma: LUAD: lung adenocarcinoma; LUSC: lung squamous cell carcinoma; MESO: mesothelioma; OV: ovarian serous cystadenocarcinoma: PAAD: pancreatic adenocarcinoma; PCPG: pheochromocytoma and paraganglioma; PRAD: prostate adenocarcinoma; READ: rectal adenocarcinoma; SARC: sarcoma; SKCM: skin cutaneous melanoma; STAD: stomach adenocarcinoma; TGCT: testicular germ cell tumors; THCA: thyroid carcinoma; THYM: thymoma; UCEC: uterine corpus endometrial carcinoma; UCS: uterine carcinosarcoma; UVM: uveal melanoma.

The CMTM1–CMTM4 genes are highly expressed in the male reproductive system (testis) and compartments in the immune system, including the bone marrow and peripheral blood cells, such as resting CD19^+^ cells and activated peripheral blood monocytes^20,26,27^. The CMTM3 and CMTM5 genes, as well as the CMTM7 and CMTM8 genes^18,28^, are broadly expressed in normal adult and fetal tissues but show decreased expression with frequent DNA methylation in the promoter regions in most carcinoma cell lines^9,29^. CMTM6 has been shown to be upregulated in the tissues of some tumors, including gliomas^17,12,30^. The pan-cancer expression of CMTM family genes and their prognostic value in The Cancer Genome Atlas (TCGA) database are summarized in **Figure 2**.

**Figure 3:**
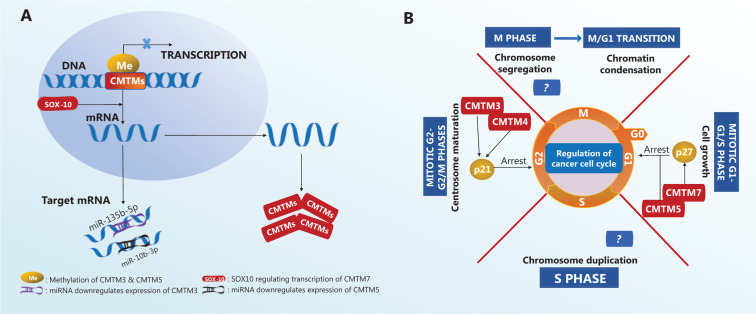
Regulatory mechanisms of CMTM expression and effects on the cell cycle in tumors. (A) CMTM expression is regulated by methylation, SOX-10, and micro-RNAs. (B) CMTM family proteins cause cell cycle arrest.

Many biological processes and molecules, such as DNA methylation and microRNAs (**Figure 3A**), regulate the expression of CMTM family members. The CMTM3 gene locus contains a typical CpG island, which is methylated to maintain gene silencing in carcinoma cell lines, including those from breast, esophageal^8^, colorectal^31^, and laryngeal squamous cell carcinomas^29^. In addition, CMTM5 gene inactivation by CpG methylation has been observed in various carcinoma cell lines, such as those from oral squamous cell carcinoma^32^, breast carcinoma^9^, and myeloid leukemia^7^. A recent study has shown that SOX10, a member of a highly conserved transcription factor family regulating cell differentiation and tissue formation, is also a potential regulator of CMTM family member expression. For example, SOX10 promotes CMTM7 expression (**Figure 3A**) in gastric cancer cells. CMTM7 knockdown promotes tumor growth, whereas SOX10-dependent CMTM7 overexpression decreases the cancer cell proliferation rate, thus slowing tumor growth^33^.

MicroRNAs are another important regulator of CMTM gene expression. In the gastric cancer cell line SGC-7901, miR-135b-5p expression is negatively correlated with CMTM3 expression (**Figure 3A**), and CMTM3 expression is markedly increased when miR-135b-5p is inhibited^34^. miR-10b-3p downregulates the expression of CMTM5 (**Figure 3A**), and lower CMTM5 expression is associated with poor prognosis in patients with hepatocellular carcinoma (HCC)^35^. High expression of miR-10b-3p is frequently detected in various types of carcinomas^36–39^, thus suggesting that further investigation of the miR-10b-3p/CMTM5 signaling axis is of high interest. The expression of CMTM family members in various cancer types and their functions in different tumors are summarized in **Table 2**.

## Signaling mediated by CMTM family in tumor biological processes

### The CMTM family regulates cancer proliferation *via* cell cycle

The cell cycle is a complex process comprising an interphase including the G1, S, and G2 phases, and a mitotic (M) phase. The cell cycle is finely regulated by a complex signaling network known as the cell cycle control system, whose prominent components are cyclin proteins and cyclin-dependent kinases (CDKs)^58^. Restriction points, known as checkpoints, are imposed at the G1-S phase and G2-M phase transitions to ensure the accuracy of DNA replication. Cyclin-CDK complexes control cell cycle progression by targeting these checkpoints. Some CDK-interacting proteins, including CIP/KIP family proteins, such as p21 and p27, are also important regulators in the cell cycle control system^59^. Dysregulation of the cell cycle leads to ectopic cell proliferation in cancers; therefore, components of cell cycle control have become therapeutic targets for cancers^59,60^. In this section, we summarize the mechanisms by which CMTM family regulates the cell cycle.

Diminished levels of CMTM3 mRNA are frequently found in urogenital cancer cell lines^44^. Reintroducing CMTM3 gene expression in a human seminoma cell line (NCCIT) by delivering an adenovirus (Ad-CMTM3) has been found to result in inhibition of cell growth and migration^44^. Furthermore, Ad-CMTM3-infected NCCIT cells express higher levels of p21, thus leading to cell cycle arrest at the G2 phase^44^. Similar effects have been observed for CMTM4 (**Figure 3B**), which has 2 alternative splicing forms, CMTM4_v1 and CMTM4_v2. This protein inhibits HeLa cell growth by inducing G2-M phase cell cycle arrest *via* a mechanism that is unclear but is interestingly not *via* apoptosis^24^. Similar regulation of the cell cycle also occurs in the clear cell renal cell carcinoma cell line 786-O, in which CMTM4 gene expression is lower than that in normal tissue, and restoration of CMTM4 expression upregulates p21 expression at both the protein and mRNA levels^23^. Other CMTM family proteins regulate the cell cycle through a distinct mechanism. CMTM5 expression is low in renal cell carcinoma (RCC), metastatic renal cell adenocarcinoma, metastatic clear renal cell adenocarcinoma, and the human kidney cancer line HK2^57^. Restoration of CMTM5 expression induces G0-G1 phase cell cycle arrest rather than G2 phase arrest (**Figure 3B**), thus decreasing the proliferation of RCC cells^57^. In addition, the CMTM7 induces G1-S phase cell cycle arrest *via* p27 (**Figure 3B**) in the nasopharyngeal carcinoma cell lines YSE410 and KYSE180.

### The CMTM family regulates endocytic EGFR levels and EGFR-induced signaling pathways

Epidermal growth factor receptor (EGFR) signaling regulates epithelial tissue development and homeostasis and is increasingly recognized as a biomarker of resistance in tumors^61^. When stimulated by epidermal growth factor (EGF), EGFR is phosphorylated, thus leading to activation of the intracellular signaling cascade that controls cell proliferation, differentiation, and migration. EGFR signaling is initiated at the plasma membrane and is negatively regulated by the internalization, ubiquitination^62^, and subsequent degradation of EGFR. In this section, we discuss how CMTM family proteins participate in regulating EGFR signaling.

**Figure 4:**
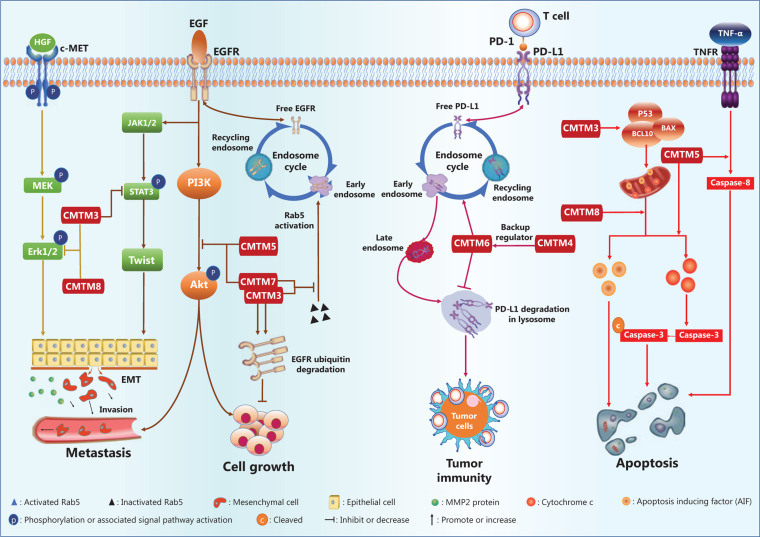
The CMTM family regulatory network in cancers.

The gastric cancer cell lines AGS and SGC-790 are defective in CMTM3 gene expression, as compared with that in normal tissue. Reintroduction of CMTM3 inhibits EGF-mediated cell migration^45^. Further mechanistic study has revealed that CMTM3 downregulates the cell surface EGFR protein level by promoting early endosome fusion *via* enhancing Rab5 activation (**Figure 4**), thus regulating EGFR endocytic trafficking^45^.

Interestingly, CMTM5 also regulates endocytic trafficking of EGFR. In addition, in HCC and prostate cancers, the expression of CMTM5 is negatively associated with activation of the PI3K/AKT pathway (**Figure 4**)^48,55^, which is downstream of EGFR signaling, thus suggesting that CMTM5 may inhibit tumor growth and metastasis by targeting AKT^48,55^. Moreover, a recent study has reported that CMTM5-v1, an alternative splicing protein isoform of CMTM5, may promote the response sensitivity of prostate cancer cells to Gefitinib, a tyrosine kinase inhibitor targeting the EGFR^56^. CMTM7, a defined tumor suppressor, has been found *via* tissue microarray analysis to be downregulated in various tumors, such as esophageal, gastric, pancreatic, hepatic, lung, and cervical tumors, as compared with the expression in normal tissues^28^. Mechanistically, ectopic CMTM7 expression inhibits EGFR-induced migration of esophageal cancer cells, similarly to the effects of CMTM3 and CMTM5 (**Figure 4**). CMTM7 has also been reported to inhibit EGFR recycling by activation-induced endocytosis, thus decreasing cell surface EGFR levels and suppressing the downstream PI3K/AKT signaling pathway (**Figure 4**), without altering ERK activation. CMTM5 and CMTM7 cooperatively promote EGFR internalization and suppress the EGFR-mediated PI3K/AKT signaling pathway^28^. Furthermore, CMTM7 and EGFR partially colocalize within the Golgi apparatus; in addition, CMTM7, like CMTM3, attenuates EGFR trafficking by enhancing Rab5 activation, thus leading to accelerated early endosome fusion, and promotes proteasomal degradation of EGFR *via* ubiquitination (**Figure 4**)^50^. Inhibition of EGFR signaling has also been observed in HL-60 cells when CMTM8 expression is restored by adenoviral infection^63^.

### The CMTM family regulates tumor invasion and metastasis by controlling epithelial to mesenchymal transition (EMT)

EMT is a program that plays key roles in embryogenesis and wound healing^64^. In cancers, activation of EMT promotes cancer progression and metastatic ability. The central regulatory network of the EMT process involves a group of regulators termed EMT-inducing transcription factors (EMT-TFs), among which Slug, Snail, Twist, and Zeb1 are prominent^65,66^. In cancer, the EMT phenotype is also mediated by a series of cell-cell signaling pathways, such as the Wnt, TGF-β, insulin growth factor, and hepatocyte growth factor (HGF) signaling pathways^65,66^. In this section, we discuss the regulatory effect of the CMTM family on EMT-TFs and EMT-related signaling.

Numerous studies have indicated that CMTM family gene expression correlates with markers of the EMT process^49,67^ and have revealed that mechanisms driving EMT are mediated by CMTM family proteins during cancer cell metastasis^40,47,49^. In the gastric cancer cell lines GES-1, AGS, and SGC-7901^67^, overexpression of CMTM3 results in downregulation of the epithelial protein E-cadherin but upregulation of mesenchymal proteins such as N-cadherin, vimentin, and MMP-2. Twist1, a transcription factor driving EMT, is significantly downregulated by overexpression of CMTM3, whereas Zeb1 and Snail are not^67^. Additionally, silencing of the CMTM3 gene increases phosphorylation of ERK1/2 (Thr202/Tyr204) and STAT3 (Tyr705) but not AKT (Ser473) (**Figure 4**) in gastric and prostate cancer cells^54,67^. Increased activity of the ERK1/2 signaling pathway has been determined not to be responsible for enhanced cell migration^67^. Instead, CMTM3 gene expression suppresses gastric cancer cell migration by regulating the STAT3/Twist1/EMT pathway (**Figure 4**)^67^. Another study has demonstrated that CMTM3 gene overexpression inhibits the metastatic capability of HCC cells, decreases the number of invading cells in Transwell migration assays, and results in increased expression of an epithelial cell marker (E-cadherin) coupled with decreased expression of mesenchymal cell markers (N-cadherin and vimentin), thus suggesting inhibition of the EMT process^47^.

CMTM8 gene expression is negatively correlated with tumor cell invasion/metastasis and markers of EMT in HepG2 cells^49^. Targeted inhibition of CMTM8 gene expression by small interfering RNAs induces morphological changes in HepG2 cells from a tissue epithelium-associated shape to a fusiform shape, inhibits ERK signaling, decreases the level of E-cadherin, and increases the protein levels of Zeb1 as well as fibronectin^49^, thus providing strong evidence that CMTM8 gene expression inhibits the EMT process. Notably, EMT inhibition in hepatocytes by CMTM8 is independent of EGFR-ERK signaling, although CMTM8 gene expression decreases EGFR levels on the plasma membrane by enhancing endocytic degradation of EGFR^63,68^. Recent evidence also indicates that CMTM8 gene expression suppresses ERK-MAPK pathway activation *via* the HGF/c-MET/ERK signaling pathway (**Figure 4**) in hepatocytes^49^.

### The CMTM family regulates cancer cell apoptosis

Apoptosis is a form of programmed cell death. Dysregulation of apoptosis influences multiple features of cancer development and progression, including cell immortalization, proliferation, and chemotherapeutic resistance^69^. Apoptosis can be triggered by the caspase-dependent extrinsic or intrinsic pathways^69^, and partially by the caspase-independent pathway^70,71^. The caspase-mediated intrinsic pathway is triggered by mitochondrial membrane potential changes due to increased levels of proapoptotic Bcl-2 family proteins (Bax and Bak) and/or decreased levels of antiapoptotic proteins (Bcl-2 and Bcl-xL). This pathway subsequently causes cytochrome c release and apoptosome assembly, thus leading to sequential activation of initiator caspases (caspase-2, -8, -9, and -10) and executor caspases (caspase-3, -6, and -7)^69^. The extrinsic pathway is initiated by activation of tumor necrosis factor receptor family proteins on the cell surface, which in turn triggers activation of caspase-8 and/or -10, thus leading to activation of downstream effector caspases-3, -6, and -7^69^. In this section, we discuss how the CMTM family regulates both caspase-dependent and caspase-independent pathways.

A recent study has reported an association between CMTM1_v5, an alternatively spliced protein isoform of CMTM1, and apoptosis in lymphoma cells; the interaction between CMTM1_v5 and calcium-modulating cyclophilin ligand negatively regulates the Ca^2+^ response in the endoplasmic reticulum in lymphoma cells and thereby results in cell apoptosis^51^. The CMTM3 gene is silenced by CpG methylation in the human nasopharyngeal carcinoma cell line CNE2, and this event is critical in promoting cancer cell survival and growth^8^. CMTM3 overexpression induces CNE2 cells to become apoptotic with enhanced caspase 3 activity^8^. In addition, CMTM3 gene expression promotes testicular cancer cell apoptosis by inducing the expression of proapoptotic proteins, including P53, APAF1, BAX, BCL10, caspase-9, and caspase-3 (**Figure 4**), and inhibits testicular cancer cell growth^44^.

Similarly to the CMTM3 gene, the CMTM5 gene is silenced with CpG methylation in the promoter region in the pancreatic cancer cell line MIA PaCa-2. CMTM5 gene expression induces apoptosis in pancreatic^53^, cervical^21^, and papillary RCC cell lines^57^. Ectopic restoration of CMTM5 expression with an adenoviral vector not only induces cell morphological changes, as discussed above, but also enhances apoptosis, as indicated by DNA fragmentation, *via* both the extrinsic and intrinsic apoptosis pathways^53^. Restoration of CMTM5 gene expression synergizes with TNF-α in activating caspase-8^53^, and induces mitochondrial transmembrane potential changes and cytochrome c release, thus promoting apoptosome assembly and caspase-9 activation in HeLa and SiHa cells^21^. However, a pan-caspase inhibitor reverses apoptosis in 90% of apoptotic cells^21^. Thus, apoptosis induced by CMTM3 and CMTM5 depends mainly on the caspase-mediated pathway (**Figure 4**).

CMTM8 gene expression also induces caspase activation and apoptosis in HeLa cells, but inhibition of caspase family proteins does not completely rescue the cells from apoptosis. CMTM8 triggers translocation of Apoptosis-Inducing Factor (AIF) from the mitochondrial intermembrane space to the cytosol and nucleus, thus inducing caspase-independent peripheral chromatin condensation and causing large-scale DNA fragmentation^43^. Both small interfering RNAs targeting AIF and pan-caspase/caspase-9 inhibitors significantly decrease the apoptosis of cells overexpressing the CMTM8 gene^43^. Therefore, CMTM8 activates both the caspase- and AIF-dependent apoptosis pathways (**Figure 4**)^25^.

### The CMTM family and tumor immunity

Immuno-oncology research is increasingly being recognized for its expanding clinical applications. The consensus is that the immune system plays an important role in tumor development; thus, immune factors can be targeted for cancer therapy or measured as biomarkers for therapeutic responses and prognosis. Immune checkpoint blockade therapies, such as anti-PD-1 therapy, induce robust and durable clinical responses in multiple cancer types^72,73^, but most patients are unresponsive to treatment. Thus, understanding additional factors that may influence therapeutic outcomes is important. The effects of CMTM family proteins in immunobiology have been reported for multiple family members. CMTM6, a CMTM family protein with previously unknown function, has been identified as a regulator of the PD-L1 protein in 2 recent studies^12,13^.

In these studies, CMTM6 has been found to stabilize PD-L1 on the cell surface, and the level of cell surface PD-L1 decreases in the absence of the CMTM6 protein^12,13^. This effect of CMTM6 is independent of the IFN-γ signaling pathway^12,13^, a major signaling pathway that induces PD-L1 expression. CMTM6 protein interacts with the transmembrane and intracellular domains of PD-L1, thus decreasing PD-L1 degradation through the ubiquitination-proteasome pathway *via* an E3 ubiquitin ligase, STUB1. The transcription, translation, and posttranslational modification of PD-L1 is unaffected by CMTM6^12,13^. Additionally, the endocytic recycling process of PD-L1 is inhibited in the absence of CMTM6, thus promoting further PD-L1 degradation *via* the lysosomal pathway (**Figure 4**) in a process critically dependent on the Rab11 protein^13^. T cell activity is enhanced in tumors formed by melanoma cancer cells deficient in CMTM6 gene expression, as indicated by the increased production of cytokines such as IL-2^12,13^. In addition, Mezzadra et al.^12^ have revealed that CMTM4 functions as a backup regulator that stabilizes PD-L1 protein. However, whether CMTM4 and CMTM6 target the same molecular pathway is undetermined.

The immunoregulatory function of CMTM6 may extend beyond PD-L1. In a bioinformatic study using gene set variation analysis on transcriptome data from 1,862 glioma samples available from the CGGA RNA-seq, TCGA RNA-seq, CGGA microarray, GSE16011, and IVY GBM databases^17^, CMTM6 expression has been found to be positively correlated with immunosuppressive factors, such as induced T cell tolerance, cytokine synthesis and secretion, and regulatory T cell differentiation^17^. Moreover, Pearson correlation analysis has shown that CMTM6 expression levels are positively correlated with those of immune checkpoint molecules other than PD-L1, such as TIM-3 and B7-H3^17^. In the same study, CMTM6 expression has also been found to be positively correlated with inflammatory responses and somatic mutations that promote the progression of cancers^17^.

CMTM7 has been identified as a transmembrane linker between BLNK and the B cell receptor (BCR), linking IgM and BLNK on the plasma membrane, thus resulting in recruitment of BLNK to the vicinity of Syk and initiation of BLNK-mediated signal transduction^74^. CMTM7 exhibits a regulatory function specifically in B1-a cells. In CMTM7-KO mice, BCR expression on the B1-a cell surface is diminished, and serum IgM levels are lower than those in normal mice^75^. Despite the lack of direct evidence supporting the function of CMTM7 in neoplastic processes, its regulatory role in B cell function suggests a possible role in antitumor immunity.

## The potential clinical value of the CMTM family

The CMTM family has been reported to have considerable clinical value for accessing pathological stages, determining therapeutic strategies, and predicting the outcomes of many cancer types, including breast cancer, NSCLC, and gastric cancer^15,16,42^.

Expression of a functional aberrant alternatively spliced form of CMTM1, CMTM1_v17, has been detected in many types of tumor tissues, including breast, kidney, lung, liver, and ovarian cancer tissues^42^. Western blot results have confirmed that breast cancer tissues exhibit higher CMTM1_v17 expression than noncancerous mammary tissues; notably, CMTM1_v17 and CA153 are coexpressed in normal/noncancerous samples and in tumor samples^42^. In many cancer types, the elevated CMTM1_v17 level is associated with lower efficacy of cancer therapy. The effect of CMTM1_v17 on neoadjuvant chemotherapy (NAC) has been evaluated in a clinical study in a cohort of 78 patients with NSCLC previously treated with NAC and surgery. The expression level of CMTM1_v17 was positively correlated with higher pathological staging and lower partial response rates, thus suggesting that CMTM1_v17 expression may cause chemoresistance^15^. Moreover, Cox regression analysis indicated that expression of CMTM1_v17 in tumor tissue after NAC is an independent prognostic risk factor, and patients with high expression have poorer overall and disease-free survival outcomes than those with low expression^15^.

Other CMTM family genes also have good prognostic value in various cancer types. Guo et al.^46^ have identified that CMTM2 is downregulated in HCC tissues *via* immunohistochemistry; according to their study, the CMTM2 expression detected by immunohistochemistry may correlate with the tumor grade and prognosis of patients with HCC. Patients lacking CMTM3 expression have poorer prognoses in gastric cancer^16^, and CMTM3 expression is a significant independent positive prognostic factor^16^. Similar results have been reported in patients with oral squamous cell carcinoma. In one study, a total of 201 tumor samples were collected *via* surgical resection, and analysis showed that CMTM3-positive patients had better prognoses than CMTM3-negative patients^52^. Moreover, CMTM3 expression levels were significantly associated with more advanced TNM stage and recurrence^52^. In addition, the frequency of CpG methylation in the CMTM3 gene promoter was a potential prognostic factor^8,29,67^, and CMTM3 hypermethylation (with the median methylation level set as the cutoff value) predicted poor overall survival in male patients with laryngeal squamous cell carcinoma^29^.

The CMTM4 gene is located on chromosome 16q22.1, a locus including numerous tumor suppressor genes. CMTM4 is the most conserved member of the CMTM family. Its tumor-suppressive effect has been identified in HeLa cells and clear cell renal cell carcinoma cell lines^23,24^. In a clinical study on a cohort of 75 HCC patients, CMTM4 has been determined to be associated with clinicopathological features and patient prognosis in HCC^76^. In one study, CMTM4 expression was found specifically in cancerous cells in 21 (28.00%) of 75 cases, but 49 (65.33%) of the 75 adjacent nontumor tissues, in agreement with its tumor-suppressive effect. The level of CMTM4 expression was negatively correlated with the tumor size, the clinical (TNM) stage and the presence of metastasis^76^. Furthermore, Kaplan-Meier and Cox regression analyses showed that negativity for CMTM4 expression is a poor prognostic factor in HCC^76^. A bioinformatic study has indicated that patients with high CMTM6 expression have poorer overall survival outcomes than those with low CMTM6 expression^17^. Moreover, CMTM6 is expressed differentially in tumors with different WHO grades and histopathologies in various malignancies. Analysis has suggested that decreased CMTM6 expression is positively correlated with IDH gene mutation^17^. CMTM6 has also been found to be a potential immunotherapy biomarker. In a retrospective study, CMTM6 has been identified as an independent factor predicting response to anti-PD-1 immunotherapy in patients with NSCLC^77^. Another study has revealed that high CMTM6 and PD-L1 co-expression is significantly associated with better overall survival in patients with NSCLC treated with anti-PD-1 immunotherapy, and quantitative assessment suggests that the prognostic value of CMTM6 may rely on its expression in stromal immune cells rather than tumor cells^78^. Given the participation of CMTM4 and CMTM6 in the biological process of PD-L1 stabilization^12,79^, the association between these 2 genes and their role in predicting immunotherapy outcomes are worthy of further investigation.

Limited clinical evidence of the direct tumor suppressor functions of CMTM5, CMTM7 and CMTM8 has been presented to date. However, recent evidence indicates that elevated miR-10b-3p/CMTM5 signaling axis activity might predict poorer overall survival in HCC patients^35^. In addition, analysis of 84 patients with bladder carcinoma has shown that low CMTM8 expression is predictive of poorer disease-free survival and overall survival outcomes^18^. Additionally, low expression of CMTM8 is correlated with higher tumor grades and TNM stages in bladder cancer^14^.

In summary, CMTM family proteins have potential value as new biomarkers for predicting prognosis and therapeutic efficacy. However, a mature promoter or inhibitor of CMTMs has yet to be developed. In 2019, Tu et al.^80^ developed a PD-L1 antibody called H1A, which promotes PD-L1 degradation *via* abrogating the interaction of PD-L1 with CMTM6. Nevertheless, the efficacy of H1A remains to be validated in clinical trials. Broadly, there is a dearth of clinical studies testing specific promoters or inhibitors targeting CMTMs. Therefore, further studies are needed to establish standards and to facilitate development of associated drugs for clinical applications.

## Future perspectives

In 2011, Douglas Hanahan and Robert A. Weinberg proposed the next generation of 10 hallmarks of cancer, which include 6 traditional hallmarks—1) sustained proliferative signaling, 2) evasion of growth suppressors, 3) resistance to cell death, 4) replicative immortality, 5) induction of angiogenesis, and 6) activation of invasion and metastasis—along with 4 new hallmarks—7) regulation of cellular energetics, 8) avoidance of immune destruction, 9) tumor-promoting inflammation, and 10) genome instability and mutation^81^. CMTM family proteins perform critical functions in many of these biological processes, such as sustaining proliferative signaling, resisting cell death, and activating invasion and metastasis^28,45,82^.

CMTM6 has been identified to regulate endocytic recycling and degradation of PD-L1^12,13^. This is the first known function of CMTM6, the first CMTM implicated in tumor immune evasion. CMTM4 may provide a compensatory mechanism when CMTM6 malfunctions. To date, no study has reported other CMTM family members in the context of antitumor immunity. Common molecular mechanisms may be involved in CMTM6-mediated regulation of PD-L1 and CMTM3/CMTM7-mediated regulation of EGFR, possibly with the participation of different Rab proteins (**Figure 4**). These findings suggest that CMTMs regulate the recycling process of various membrane proteins with precise target specificity^12,13^, although common mechanisms may be involved. Currently, the questions of how many proteins are specifically targeted by CMTMs and what their common molecular characteristics are remain to be answered.

The roles of CMTMs in tumor development and metastasis have been widely studied since the discovery of this protein family, but to understand the molecular mechanisms underlying the regulation of CMTM expression, many details require further investigation. In summary, CMTM3, CMTM4, CMTM5, CMTM7, and CMTM8 exhibit inhibitory effects on the biological processes of tumor cells^11,16,21–25,28,43–45,48–50,55,57,83^. However, CMTM1 and CMTM6 may act as oncogenes in many solid tumors^12,13,15,17,30,41,42^. DNA methylation is the dominant mechanism in silencing CMTM3 and CMTM5 gene expression in cancer cells, and microRNAs also play an important role, although these regulatory paradigms have not been reported for other CMTMs. In addition, genetic mutation analysis data of CMTM genes in tumor cells are lacking. The function of CMTM2 in cancer remains unclear.

Genetic heterogeneity among cancer types and within individual cancer patients poses further challenges in translating research results into clinical practice. Although the data from model systems suggest an important role of CMTMs in cancer development, to date, most clinical studies have reported only the prognostic value of CMTM family genes, and there is no evidence indicating whether aberrant CMTM expression directly interferes with therapies, including chemotherapy, radiotherapy, and immunotherapy. Additionally, the influence of CMTMs on long-term outcomes is unknown. We anticipate that this review will incite further interest in investigating the functions of CMTMs to promote clinical translation.

## Figures and Tables

**Table 1:** Characteristics of CMTM family members

Gene	Location	Main subcellular locations	Gene ontology (GO) - biological process
CMTM1	16q21	Plasma membrane, extracellular space, nucleus, peroxisome	Chemotaxis, regulation of signaling receptor activity
CMTM2	16q21	Nucleus, plasma membrane, extracellular space, Golgi apparatus, peroxisome, cytosol	Chemotaxis, regulation of signaling receptor activity
CMTM3	16q22.1	Plasma membrane, extracellular space, nucleus, endosome	Chemotaxis, regulation of signaling receptor activity, positive regulation of B cell receptor signaling pathway
CMTM4	16q22.1	Plasma membrane, extracellular space, nucleus, Golgi apparatus	Chemotaxis, regulation of signaling receptor activity
CMTM5	14q11.2	Plasma membrane, extracellular space	Chemotaxis, regulation of signaling receptor activity, negative regulation of myoblast differentiation
CMTM6	3p22.3	Plasma membrane, extracellular space, lysosome, cytoskeleton, cytosol, endosome	Chemotaxis, regulation of signaling receptor activity, neutrophil degranulation
CMTM7	3p22.3	Plasma membrane, extracellular space	B-1a B cell differentiation, chemotaxis, regulation of signaling receptor activity
CMTM8	3p22.3	Nucleus, plasma membrane and extracellular space	Membrane raft polarization, chemotaxis, protein localization, regulation of signaling receptor activity, myelination

The information is available in the Gene Cards database: https://www.genecards.org/

**Table 2:** Expression and functions of CMTM family members in different tumors

Cancer types	Experimental models	Expression in tumors	Functions	Refs
Bladder				
CMTM3	Cell lines	–	↓ AR transactivation, ↓ PSA expression	^40^
CMTM8	Cell lines	–	↓ growth, ↓ migration and invasion, ↑ apoptosis	^14,18^
Brain				
CMTM3	Human tumor tissue	High	↑ invasion	^41^
CMTM4	Human tumor tissue	Low	–	^41^
CMTM6	Human tumor tissue	High	–	^41^
CMTM8	Human tumor tissue	Low	–	^41^
Breast				
CMTM1	Human tumor tissue, cell lines	High	↑ proliferation, ↓ apoptosis	^42^
CMTM3	Cell lines	Low	↓ growth	^8^
CMTM5	Cell lines	Low		^9^
CMTM8	Cell lines	–	↑ apoptosis	^43^
Cervical				
CMTM4	Cell lines	–	↓ growth, ↑ cell cycle arrest	^44^
CMTM5	Cell lines, human tumor tissue	Low	↓ colony formation, ↑ apoptosis	^6,9,21^
CMTM7	Cell lines, human tumor tissue	Low		^28^
CMTM6	Cell lines	–	↑ immune evasion	^12^
Colorectal				
CMTM3	Cell lines	Low		^8^
CMTM4	Cell lines	–	↑ immune evasion	^12^
CMTM5	Cell lines	Low		^9^
CMTM6	Cell lines	–	↑ immune evasion	^12^
CMTM8	Cell lines	Low		^10^
Esophageal				
CMTM3	Cell lines	Low	↓ growth	^8^
CMTM5	Cell lines	Low	–	^9^
CMTM7	Cell lines, human tumor tissue	Low	↓ growth, ↑ cell cycle arrest	^28^
CMTM8	Cell lines	Low	–	^10^
Gastric				
CMTM3	Cell lines, nude mice	Low	↓ growth, ↓ migration and invasion, ↓ peritoneal metastasis (in mice), ↑ apoptosis, ↑ cell cycle arrest	^8,16,34,45^
CMTM7	Cell lines, human tumor tissue, nude mice	Low	↓ growth, ↓ migration and invasion	^28,33^
CMTM8	Cell lines	Low		^10^
				
Hematological				
CMTM1	Cell lines	High	–	^42^
CMTM5	Cell lines	Low	↓ growth	^7^
Liver				
CMTM1	Human tumor tissue	High	–	^42^
CMTM2	Human tumor tissue	Low	–	^46^
CMTM3	Cell lines	Low	↓ growth, ↓ migration and invasion	^47^
CMTM5	Human tumor tissue, cell lines, nude mice	Low	↓ growth, ↓ migration and invasion	^9,35,48^
CMTM8	Cell lines, human tumor tissue	Low	↓ migration and invasion	^10,49^
Lung				
CMTM4	Cell lines	–	↑ immune evasion	^12^
CMTM5	Cell lines	Low	–	^9^
CMTM6	Cell lines	–	↑ immune evasion	^12^
CMTM7	Cell lines, human tumor tissue	Low	↓ growth, ↓ migration and invasion	^28,50^
Lymphoma				
CMTM1	Cell lines	-	↑ apoptosis	^51^
Melanoma				
CMTM4	Cell lines	–	↑ immune evasion	^12^
CMTM6	Cell lines	–	↑ immune evasion	^12,13^
Nasopharyngeal				
CMTM3	Cell lines	Low	↓ growth, ↑ apoptosis	^8^
CMTM5	Cell lines	Low	–	^9^
Oral				
CMTM3	Cell lines, nude mice	Low	↓ growth, ↓ migration,	^52^
CMTM5	Cell lines	Low	↓ growth, ↓ migration and invasion, ↑ apoptosis	^32^
Ovarian				
CMTM1	Human cancer tissue, cell line	High	–	^42^
CMTM5	Human cancer tissue	Low	–	^6^
Pancreatic				
CMTM5	Human cancer tissue, cell line	Low	↑ apoptosis	^53^
CMTM7	Cell lines, human tumor tissue	Low	–	^28^
Prostate				
CMTM3	Cell line	Low	↓ growth, ↓ migration and invasion	^8,54^
CMTM5	Cell lines	Low	↓ growth, ↓ migration and invasion, ↑ response sensitivity to Gefitinib	^9,55,56^
Renal				
CMTM1	Human tumor tissue	High	↑ apoptosis	^42^
CMTM3	Human tumor tissue, cell lines	Low	↓ growth, ↓ migration and invasion	^11^
CMTM4	Human tumor tissue, cell lines	Low	↓ growth, ↓ migration and invasion, ↑ cell-cycle arrest, ↑ apoptosis	^23^
CMTM5	Human tumor tissue, cell lines	Low	↓ growth, ↓ migration and invasion, ↑ cell-cycle arrest, ↑ apoptosis	^57^
Testicular				
CMTM3	Cell lines	Low	↑ cell-cycle arrest, ↑ apoptosis, ↓ growth and migration	^44^

↑: promotes, ↓: inhibits, -: not reported.
